# Managing Severe Cancer Pain with Oxycodone/Naloxone Treatment: A Literature Review Update

**DOI:** 10.3390/jpm14050483

**Published:** 2024-05-01

**Authors:** Paolo Formenti, Michele Umbrello, Mauro Pignataro, Giovanni Sabbatini, Lorenzo Dottorini, Miriam Gotti, Giovanni Brenna, Alessandro Menozzi, Gaetano Terranova, Andrea Galimberti, Angelo Pezzi

**Affiliations:** 1 SC Anestesia, Rianimazione e Terapia Intensiva, ASST Nord Milano, Ospedale Bassini, Cinisello Balsamo, 20097 Milan, Italy; 2 Department of Intensive Care, New Hospital of Legnano (Ospedale Nuovo di Legnano), 20025 Legnano, Italy; 3 Contenuto Ed Net Communications SL, 20131 Milan, Italy; 4 Oncology Unit, ASST Bergamo Ovest, 20047 Treviglio, Italy; 5 School of Medicine and Surgery, University of Milano-Bicocca, 20126 Milano, Italy; 6 Anaesthesia and Intensive Care Department, Asst Gaetano Pini, 20100 Milano, Italy

**Keywords:** severe cancer pain 1, oxycodone/naloxone 2, pain management 3

## Abstract

Severe cancer pain substantially affects patients’ quality of life, increasing the burden of the disease and reducing the disability-adjusted life years. Although opioid analgesics are effective, they may induce opioid-induced bowel dysfunction (OIBD). Oxycodone/naloxone combination therapy has emerged as a promising approach to mitigate opioid-induced constipation (OIC) while providing effective pain relief. This review provides an updated analysis of the literature of the last decade regarding the use of oxycodone/naloxone in the management of severe cancer pain. Through a comprehensive search of databases, studies focusing on the efficacy, safety, and patient experience of oxycodone/naloxone’s prolonged release in severe cancer pain management were identified. Furthermore, the literature discusses the mechanism of action of naloxone in mitigating OIC without compromising opioid analgesia. Overall, the evidence suggests that oxycodone/naloxone combination therapy offers a valuable option for effectively managing severe cancer pain while minimizing opioid-induced constipation, thereby improving patients’ quality of life. However, further research is needed to optimize dosing regimens, evaluate long-term safety, and assess patient outcomes in diverse cancer populations.

## 1. Introduction

Cancer is a widespread disease affecting millions of patients globally, resulting in physical, emotional, and psychological distress [1]. Despite recent investigations indicating a decrease in both the prevalence and severity of cancer pain, nearly half of all cancer patients still experiences pain [2]. Effectively managing severe pain poses a significant challenge, as it can substantially impact the patient’s quality of life, resulting in higher dependency on caregivers [3]. Relief from pain can be achieved through various medications, with opioid analgesics considered the gold standard for treating severe cancer pain. Nevertheless, opioids can cause some significant side effects named Opioid-induced bowel dysfunction (OIBD), which may include nausea, vomiting, drowsiness, and constipation [4]. OIDB poses a substantial challenge for both patients and healthcare providers, as it not only leads to physical discomfort and reduced quality of life, but also affects treatment adherence and patient satisfaction. In recent decades, an oral pharmaceutical formulation with a fixed 2:1 ratio combination of oxycodone and naloxone in prolonged released (PR) has been introduced to address this challenge providing analgesia by binding to opioid receptors in the central nervous system, and simultaneously acting as a peripheral antagonist targeting opioid receptors in the gastrointestinal system [5]. Even though the topic has been previously discussed [6,7], a careful analysis of the literature reveals that a targeted approach in severe cancer pain patients alone is still lacking. First, numerous attempts have been made to categorize cancer patients based on the intensity of their pain, serving both clinical and research objectives. One such classification system, based on numeric rating scale (NRS), identified severe pain once it has been reported between 7 to 10 [8]. However, it is worth noting the inherent challenge in effectively differentiating between moderate and severe cancer pain, as described, since subjective interpretation, varying pain thresholds among patients, and the multifaceted nature of pain perception contribute to this complexity [9]. As a result, many studies encounter challenges in precisely defining pain levels to distinguish between moderate and severe categories. To address these challenges and potentially increase case numbers, researchers often combine these two patient groups. This amalgamation is evident in the limited number of references available, as studies tend to encompass both moderate and severe pain cases. Given the implication of managing severe cancer pain and the potential benefits of oxycodone/naloxone PR in addressing the challenges of opioid-induced constipation, this review aims to provides an updated analysis of the literature focusing on the efficacy, safety, patient experience, and overall impact of oxycodone/naloxone PR in the management only in severe cancer pain. A systematic search of the following databases was undertaken PubMed, Cochrane Library, Scopus, and Web of Science, covering the period from their inception to January 2024. Various combinations of keywords such as “oxycodone/naloxone”, “oxycodone/naloxone prolonged release (PR)”, “severe cancer pain”, “opioid cancer pain treatment”, “chronic cancer pain”, “efficacy”, “safety”, “oxycodone/naloxone PR and sides effects”, and “patient experience and quality of life” were utilized with appropriate Boolean operators across these databases. Full-text articles deemed relevant were retrieved by two authors (MM and PF), while additional titles and abstracts were screened and their full versions obtained. Reference lists of included studies and review articles were manually examined to identify any further pertinent studies. Full-text documents were initially evaluated for relevance and assessed using the Critical Appraisal Skills Program (CASP) checklist. Articles failing to meet CASP’s essential criteria, such as alignment with the review’s aim, were excluded from further analysis. Additionally, a similar search was conducted using the PubMed MeSH thesaurus. Ultimately, 87 eligible studies were identified, and after independent screening and full-text review, 23 studies were included in the analysis.

## 2. Enhanced Oxycodone/Naloxone Features

**Figure 1:**
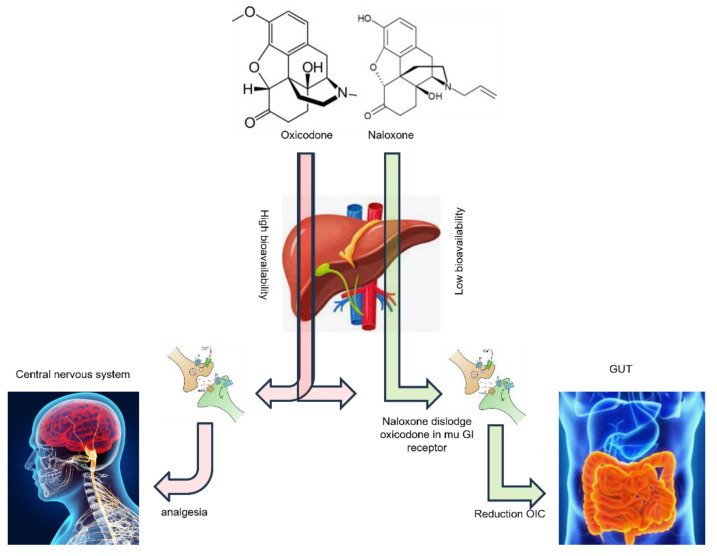
Schematic diagram of mechanism involved in Oxycodone/naloxone PR. Overall, the schematic diagram illustrates how the combination of oxycodone and naloxone in a 2:1 ratio addresses both pain relief and opioid-induced constipation by targeting specific opioid receptors in different areas of the body.

Oxycodone acts as a full opioid receptor agonist, binding to mu, kappa, and delta opioid receptors in the central nervous system and the mu receptor in gastrointestinal tract. These bindings result in analgesic effects, but can also lead to opioid-induced constipation [2]. Compared to morphine, oxycodone has a higher oral bioavailability and, following enteral absorption, it distributes throughout the body, with approximately half bound to plasma proteins. By displacing oxycodone from these receptors, naloxone effectively reverses the constipating effects of opioids without affecting the analgesic effects in the central nervous system [10]. Additionally, naloxone’s limited absorption and initial liver metabolism contribute to its low bioavailability, ensuring that its systemic effects are minimal [11]. Although diarrhea may occur initially with naloxone treatment, it tends to be transient. The oral administration of naloxone is unlikely to produce significant systemic effects due to its pronounced first-pass effect and low oral bioavailability (Figure 1). The combination of oxycodone and naloxone is often formulated in a fixed 2:1 ratio, where the amount of naloxone is approximately half that of oxycodone. This fixed ratio ensures consistent delivery of both medications and optimizes their synergistic effects in managing pain and mitigating opioid-induced constipation [12]. Moreover, oxycodone/naloxone formulations are often designed as PR formulations. This allows for a gradual release over an extended period after administration, providing sustained pain relief and minimizing the need for frequent dosing. The prolonged-release formulation also contributes to improved patient adherence and convenience, as it reduces the frequency of medication intake while maintaining therapeutic efficacy. In cases of liver dysfunction or porto-systemic shunting, which are often present in various advanced cancers, it is important to keep in mind the potential for diminished first-pass hepatic metabolism, even if liver function test results fall within the normal range. This consideration is essential to prevent unexpected adverse effects [13].

### 2.1. Cancer Pain Peculiarities

Cancer is more than just cancer cells, as it also includes a variety of surrounding cells like immune cells, mesenchymal cells, and endothelial cells. The stroma plays a crucial role in tumor development and progression by providing support and promoting interactions with surrounding tissues [14]. The relationship between the tumor and its microenvironment is dynamic and multifaceted. Both the tumor cells and the stromal cells secrete various mediators that modulate this relationship [15]. These mediators can influence processes such as angiogenesis, immune response, and tissue remodeling, ultimately shaping the tumor microenvironment and affecting tumor behavior. Interestingly, the development of many tumor types appears to involve the exploitation of host neuronal tissue [16]. Tumors can produce neurotrophic factors, such as nerve growth factor, which promote the survival and growth of nerves within and around the tumor. This phenomenon may contribute to the observed increase in neuronal density in the vicinity of tumors [17]. Moreover, tumors release a range of molecules that modulate pain perception. These molecules include hydrogen ions, inflammatory cytokines such as Tumor Necrosis Factor-alpha (TNF-α) and Interleukins (IL-1, IL-6), growth factors like Transforming Growth Factor-beta (TGF-β), and prostaglandins [18,19]. These substances sensitize and activate sensory nerve fibers, leading to the experience of pain. This localized release of pain-modulating agents contributes to the phenomenon of tumor-associated pain, which can significantly impact the quality of life of patients [20,21]. Additionally, central effects of these molecules are observed, leading to neuronal hyperexcitability and further amplification of pain signals. This central sensitization process involves changes in the excitability of neurons in the central nervous system, leading to enhanced responses to painful stimuli [22,23]. Thus, the interaction between tumors and their microenvironment is complex and involves various cellular and molecular components.

### 2.2. Varied Clinical Pain Expression among Different Types of Cancer

**Figure 2:**
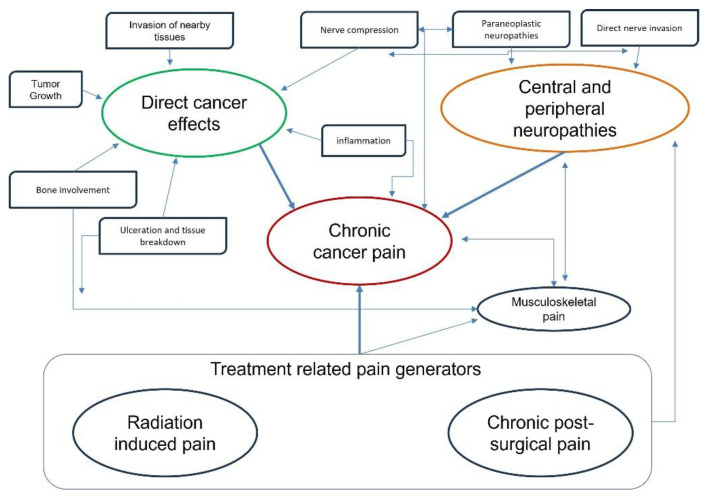
Mechanisms of cancer pain as a direct or indirect consequence of the tumor. Cancer pain arises from tissue damage, nerve compression, and inflammatory effects. This can lead to peripheral and central neuropathy, exacerbating pain severity. Additionally, medical interventions like radiation therapy can worsen the pain experience.

The sensitivity to pain varies significantly among different types of cancer. Some types of cancer may cause localized pain, such as bone cancer often resulting in sharp and persistent pain in the affected area. Other types of cancer, such as those involving internal organs, may cause more diffuse and generalized pain [20]. Furthermore, the severity of pain can also vary within the same type of cancer, depending on the stage of the disease and individual response to treatment. Among the variety of factors generators’, direct effects such as tissue ulceration, bone involvement, or invasion of nearby tissues are involved. Additionally, it can be associated with peripheral nerve involvement due to nerve compression or inflammatory effects. These processes can contribute to the development of peripheral and central neuropathy, which, in turn, may be associated with musculoskeletal involvement, leading to chronicization and increased severity of pain [24]. Furthermore, pain induced by medical or surgical interventions, such as radiation therapy or post-operative pain, can exacerbate the overall pain experience for individuals with cancer (Figure 2). It is challenging to discern from the literature the potential clinical effects of using oxycodone/naloxone PR in various types of cancer because most available studies do not stratify patients based on tumor type. However, tumor type can have different effects on the genesis of cancer pain. For instance, lung carcinoma is often associated with persistent cough, dyspnea, and bone metastases [25]. In an observational study of patients affected by lung cancer associated with neuropathic pain, more than 80% of patients significantly reduced the average pain intensity improving health-related patient-reported outcomes [26]. Almost 40% of cancer patients participating in two prospective 28-day trials, where oxycodone/naloxone PR demonstrated comparable analgesic efficacy for moderate-to-severe cancer pain, experienced lung cancer [27]. This formulation exhibited advantages over other potent opioids, including lower daily doses, reduced necessity for dose escalation, and fewer side effects. Similarly, breast cancer can cause pain from local lesions, bone metastases, or chemotherapy-induced neuropathy [28]. In a retrospective study by Cuomo et al. [29], 33 patients diagnosed of lung cancer and 36 with breast cancer treated with oxycodone/naloxone PR showed a significant decrease in pain score measured on a visual analogue scale over 28 days without adverse effects on bowel function, nor change in laxative use. In colon-rectal cancer and pancreatic cancer, abdominal pain may be localized in the lower or upper abdomen, depending on the location and size of the tumor, and in cases where the tumor causes narrowing or blockage of the intestinal lumen, the patient may experience intense abdominal pain associated with cramps and abdominal distension [30,31]. Even more reason, when opioid-based pain therapy is indicated, oxycodone/naloxone can find a valid indication for its effectiveness in reducing abdominal side effects [32]. For instance, among 176 cancer patients, approximately one-third had intestinal neoplasia, and treatment with oxycodone/naloxone demonstrated good analgesic efficacy [33]. However, this subset of patients was more prone to reduce clinical response, but showed a reduction in constipation symptoms.

## 3. The Safety and Efficacy of Oxycodone/Naloxone PR in Cancer Pain

Clinical trials comparing oxycodone/naloxone to traditional opioid regimens have consistently demonstrated comparable analgesic efficacy. Improvement in analgesic efficacy and safety profile in patients with non-malignant or malignant pain were confirmed in a phase III clinical trial [34]. A comparison between the administration of oral oxycodone/naloxone PR and transdermal fentanyl was proposed in patients with moderate-to-severe cancer pain [27]. Despite a similar analgesic activity, oxycodone/naloxone PR was characterized by lower daily dosages, less need for drug escalation, and fewer side effects. In patients with chronic cancer pain, oxycodone/naloxone PR provided an analgesic effect that was like oxycodone alone, with early and sustained benefits in tolerability [35]. The overall incidence of drug-related adverse events was 28.9% in the oxycodone group and 8.2% in the oxycodone/naloxone group, with a quality of life improved to a significantly greater extent in this group. Studies have also shown that oxycodone/naloxone maintains pain control without compromising the opioid’s analgesic effect. The analgesic response was assessed in cancer patients experiencing moderate to severe pain and receiving oxycodone/naloxone PR, demonstrating a reduction in both average and worst pain intensity over time, along with a decrease in the prevalence of breakthrough pain [33]. Furthermore, 81.3% of patients exhibited a positive response to the treatment. A randomized, double-blind, active-controlled, study investigated the efficacy of receiving up to 120 mg/day of oxycodone/naloxone PR in patients affected by moderate to severe cancer pain as compared to oxycodone alone or shifted from other opioids [36]. The authors showed how patients who were switched directly from other opioids to oxycodone/naloxone PR experienced a similar analgesic effect, with a mean BPI-SF scores comparable for the two groups and with a slight rating decrease during the observation period. 

In an open-label extension of a randomized double-blind study, 128 patients with moderate-to-severe cancer pain were randomized to receive oxycodone/naloxone PR or oxycodone alone [37]. Average pain scores based on the modified BPI-SF were low and stable over the study period. In patients with cancer pain, a randomized double-blind treatment comparison between oxycodone/naloxone PR with controlled-release oxycodone or controlled-release morphine showed how a stable analgesia was achieved by 83% of controlled-release oxycodone and 81% of controlled-release morphine patients [38]. The findings from RCTs are supported by open-label studies, including 60-day observational study patients who required oxycodone/naloxone PR at high daily doses. The results showed how compared with baseline oxycodone/naloxone PR reduced pain intensity, the impact of pain on quality of life, and the number of breakthrough pain episodes [39]. A multi-center, open-label, randomized, phase IV study is still proceeding to evaluate pain difference on BPI-SF in cancer pain patients marked by a numerical pain score of ≥4/10 at baseline [40]. The authors also evaluated the effectiveness and safety of oxycodone/naloxone PR in a subgroup of patients showing clinically relevant improvements in pain intensity both in opioid-naïve patients and in patients pretreated with weak or strong opioids. A significant improvement in pain intensity was showed in exploratory, non-randomized, open-label, mono-center study in which the principal aim was to evaluate whether patients with advanced cancer and moderate to severe cancer pain will benefit from treatment with oxycodone/naloxone PR [41]. An additional observational study reported significant improvements in pain scores [29]. 

Regarding the side effects, a recent metanalysis including thirteen studies, indicated that naloxone could significantly reduce the occurrence of nausea, and vomiting induced by opioids, without relieving pain and somnolence [42]. The oxycodone/naloxone’s efficacy is represented also by its potential to reduce the need for additional laxatives. In fact, by mitigating the occurrence of OIC, patients can avoid the use of adjunct medications [4]. A multicenter study reported the effectiveness of oxycodone/naloxone PR in patients with severe pain who had laxative refractory OIC with their previous opioid [43]. The results showed how more than 60% of patients reported both an improvement in constipation and in quality of life. In summary, oxycodone/naloxone PR has demonstrated considerable efficacy in providing pain relief for cancer patients experiencing severe pain. The combination’s ability to effectively address opioid-induced constipation while maintaining analgesic potency represents a significant advancement in cancer pain management. In the latest multi-center, randomized, double-blind controlled trial, improvements in bowel function were observed numerically and statistically confirmed by a post hoc analysis [44]. This trial involved 232 cancer patients experiencing moderate-to-severe pain, who were treated with either oxycodone/naloxone PR or oxycodone PR for a duration of 4 weeks. The principal studies focused on oxycodone/naloxone PR in severe cancer pain are summarized in Table 1.

## 4. Patient Experience and Quality of Life

A substantial portion of individuals diagnosed with cancer and those who have survived the disease deal with various forms of social isolation, with one-third expressing recurrent feelings of loneliness [45]. In this context, the patient’s experience and quality of life (QoL) are crucial considerations in the management of severe cancer pain. Since opioids are one of the cornerstones of pain therapy in oncology patients, the potential to reduce their side effects represent a significant aspect for improving the QoL and adherence to chronic treatment. QoL is a multidimensional parameter for which coverage may be categorized within five dimensions including physical, material, social, and emotional wellbeing [46]. Unfortunately, there is a lack of uniformity in the assessment of QoL across different studies, making it challenging to draw definitive conclusions. Often, the effect of pain on patients’ Quality of Life (QoL) is assessed by considering the domains outlined in the BPI-SF [47]. These domains, including general activity, walking ability, normal work, mood, enjoyment of life, sleep, and relations with other people, are evaluated using an eleven-point (NRS). Scores range from 0 (indicating no impairment) to 10 (indicating the most severe impairment), and they are consolidated by computing the average score across these seven items. Oxycodone/naloxone has shown positive effects on patient experience, not only because it is well-tolerated and offers adequate analgesic value, but also because of its ability to alleviate OIBD [48,49,50]. A recent Cochrane literature revision assessed OIBD prevalence, and the safety of mu-opioid antagonists as compared to different dosage, alternative pharmacological/non-pharmacological interventions, or placebo [51]. The mu-opioid antagonists evaluated the effects of oral naldemedine, and naloxone taken in combination with an opioid treatment in cancer patients. Within the study, naldemedine and methylnaltrexone were compared with placebo, while naloxone was compared with a placebo or opioid treatment only. The results showed how, in 1343 patients, the evidence was very low to moderate because of the design of the studies, including under-reporting of trial methods. There was low confidence in the evidence that there was no impact from naloxone in combination with an opioid in pain relief, and there was uncertain evidence that naloxone taken with an opioid treatment improved symptom of constipation. Despite these recent observations, few papers report an improvement in Opioid-induced bowel dysfunction as defined by The Bowel Function Index (BFI), which is a clinician-administered, patient-reported, three-item questionnaire [52]. Patients treated with oxycodone/naloxone PR as compared with oxycodone alone showed improved BFI scores, constipation-related QoL assessments, and reduced laxative intake by 20% [37]. Moreover, BFI scores significantly improved from baseline, and the proportion of patients receiving laxatives and/or enemas declined in patient switched to higher doses of oxycodone/naloxone PL [39]. In patients diagnosed with OIC, oxycodone/naloxone PR was associated with significant improvements in BFI, stool consistency, spontaneous bowel movements, and Patient Global Impression [41]. In the double-blind RCT that compare oxycodone/naloxone PR vs. oxycodone alone in patients with OIC and cancer-related pain showed greater reductions in BFI, less laxative use, and more complete spontaneous bowel movement [34]. Nolte et al. [40] showed relevant improvements in bowel function, as described by the reductions in the mean BFI of −20.5 and −36.5 in patients pretreated with weak and strong opioids. Furthermore, a substantial improvement in QoL was described by the decrease of pain-related functional impairment across all domains included in the BPI-SF. Otherwise, an observational study of cancer patients treated in an outpatient setting reported no clinically significant change in BFI scores or laxative intake as compared with prior analgesic therapy [29]. The combination of pain relief and reduced OIC-related side effects contributes to improved treatment adherence. In fact, adherence to cancer pain therapy is often suboptimal due to several factors which include the fear of addiction, physiological and harmful effects, tolerance, disease progression, and opioid side effects [53]. Extensive data suggest that, in a significant number of patients undergoing opioid therapy, the decision to switch opioids is frequently driven by poor tolerability associated with bowel dysfunction, rather than inadequate pain relief. This strategy, known as opioid switching, is widely acknowledged as an effective method for optimizing pain control while mitigating the adverse effects induced by opioids [54]. Another aspect, sometimes overlooked but which can have a positive impact on both quality of life and treatment adherence, is the fact that since the medication is a combination of molecules, the total number of drugs the patient must take daily is significantly reduced [12]. There are no studies that have specifically verified this aspect, but it is reasonable to assume that the reduction in the number of medications may have a positive impact on patients’ overall treatment experience and may also result in cost savings, such as the avoidance of additional laxatives or stool softeners.

## 5. Controversy and Limitations

Despite all that has been described previously, there are some controversial aspects and limitations regarding the use of oxycodone/naloxone PR in cancer pain patients which include the individual variability, the pain sensitivity, opioid tolerance, comorbidities and concomitant medications, and physical and social factors. One of the critical factors to consider in the management of severe cancer pain with oxycodone/naloxone is the significant variability in individual responses to opioids [55]. 

The management of pain often necessitates a gradual process of trial and error. However, inquiring whether there exists a method to select the appropriate medication and dosage for a patient can lead us to consider pharmacogenomics. This emerging field involves the genetic prediction of medication response and holds promise in identifying the most suitable treatment options for individuals, potentially streamlining the process of pain management [56]. Pharmacogenomics represents a shift in our current medical approach from reactive to preventive, examining genetic variations as key factors influencing the broad spectrum of drug responses observed today. A prominent example of pharmacogenetic variability lies within the cytochrome (CY) P450 enzymes, pivotal in opiate metabolism [57]. Clinical recommendations exist to inform the therapeutic approach for several opioids based on CYP2D6 genotype, albeit with varying levels of supporting evidence for each medication [58]. Guidelines from the Clinical Pharmacogenetics Implementation Consortium offer genotype-based recommendations for oxycodone and other opioids, drawing from the available clinical evidence. Clinical Pharmacogenetics Implementation Consortium guidelines provide CYP2D6 genotype-based recommendations for oxycodone and other opioids, based on the available clinical evidence [59]. Even though, in CYP2D6 normal metabolizers patients, less than 10% of oxycodone is metabolized to oxymorphone, which have higher affinity for mu-receptor. Oxycodone is still the main contributor to pain relief [60]. Among cancer patients prescribed oxycodone, a cross-sectional multicenter study revealed no statistically significant variance in serum oxymorphone concentrations between CYP2D6 ultrarapid metabolizers and normal metabolizers [61]. Clinical studies in postoperative patients and in patients with cancer failed to demonstrate a significant difference in analgesia or adverse events to oxycodone by CYP2D6 phenotype [62]. Similarly, the presence of comorbidities and the use of other medications can affect the effectiveness of pharmacokinetics and pharmacodynamics of opioids. For example, the administration of oxycodone/naloxone PR should be approached with caution and close monitoring in patients with mild hepatic or renal impairment. In cases of moderate to severe hepatic impairment, its use is contraindicated due to the potential elevation of naloxone plasma levels, which can counteract the analgesic effects of oxycodone [13,63]. In the brief 4-week RCT already mentioned above, it was observed that oxycodone/naloxone PR exhibited comparable analgesic efficacy to PR oxycodone in cancer patients, with a concurrent alleviation of constipation symptoms [37]. The statistical significance in the reduction of the primary outcome, as measured by the change in constipation symptoms using the BFI, was noteworthy between the two groups. Although the reported difference approached borderline clinical significance, specifically a minimum of 12 points, the authors asserted its clinical relevance based on a comprehensive consideration of additional analyses. It is crucial to note that while various secondary outcome measures and analyses corroborate a reduction in constipation symptoms, the authors’ claim of clinical relevance is not unequivocally robust. The findings of this trial, despite its limited duration, align with prior investigations conducted in non-cancer patient populations. Nevertheless, akin to preceding studies, it does not offer insights into whether oxycodone/naloxone PR confers advantages over standard treatment involving strong opioids (such as modified-release morphine) and traditional prophylactic laxative interventions. After that, a prolonged exposure to opioids therapy may lead to the development of tolerance, which means that patients progressively require higher doses over time to achieve the same level of pain relief [39]. This issue may be less significant in the case of cancer pain when considered from the temporal perspective in which it is used, even if tolerance may result in diminishing efficacy and necessitate dose adjustments. Finally, multimodal analgesia should also be considered in the management of cancer pain [64]. This is an approach in which various analgesics are combined to maximize pain relief while simultaneously minimizing their adverse effects [65]. In a cancer pain setting, multimodal pain management approaches—which may include non-opioid analgesics, physical therapy, psychological interventions, and complementary therapies to balance the analgesic effects of oxycodone/naloxone PR—should be implemented to minimize the risk of opioid tolerance and dependence [66].

## 6. Conclusions

Overall, oral oxycodone/naloxone PR represents a valuable option for the management of severe cancer pain, offering a balanced approach between effective pain relief and improved gastrointestinal tolerability. Extensive clinical evidence supports its efficacy in improving bowel function and enhancing the overall quality of life for patients with severe cancer pain, although there have been few studies investigating its role in this setting over the last decade. Despite its significant benefits, careful consideration must be given to individual variability in opioid response, potential tolerance issues, and the presence of comorbidities. Additionally, the integration of pharmacogenomic information and multimodal analgesia approaches could further optimize treatment outcomes and minimize potential challenges associated with long-term opioid therapy.

## Figures and Tables

**Table 1:** Principal studies regarding the use of oxycodone/naloxone prolonged relies in severe cancer-related pain.

Study	Patients	Design	Main Findings
De Santis et al. [26]	56 patients with cancer lung and severe neuropathic pain	Open-label, 4-week observational study OXN + pregabalin	OXN PR + pregabalin resulted in better pain intensity, BPI-SF, and episodes/intensity of breakthrough pain.
Roberto et al. [27]	336 patients with moderate-to-severe	two prospective 28-day trials received either fentanyl or OXN-PR, baseline and after 7, 14, 21, and 28 days	Similar to analgesic activity in moderate-to-severe cancer pain, OXN-PR is characterized by lower daily dosages, less need for drug escalation, and fewer side effects compared to TDF.
Cuomo et al. [29]	206 patients with moderate-to-severe pain	Retrospective, single-center 28-day observational study of OXN PR	OXN PR resulted in markedly enhanced pain relief without adversely affecting bowel function (improvement in BFI).
Dupoiron et al. [34]	243 patients with OIC and cancer-related	Double-blind 5-week RCT OXN PR vs. OXY PR followed by 24-week open-label extension phase	OXN PR revealed noninferior analgesia, greater reductions in BFI, reduced laxative use, and increased CSBM.
Lazzari et al. [35]	146 opioid-naïve cancer patients with moderate-to-severe pain	Single-center, retrospective, observational, propensity matched study OXN PR vs. OXY PR	OXN PR and OXY PR demonstrated similar analgesic efficacy. The Bowel Function Index showed improvement from baseline with OXN PR. Adverse drug reactions were less frequent with OXN PR.
Ahmedzai et al. [37]	185 patients with moderate-to-severe cancer-related pain	Double-blind 4 weeks RCT OXN PR (≤ 120/60 mg/day) vs. OXY PR	OXN improved BFI scores constipation-related QoL assessments, reduced laxative intake by 20%, and provided noninferior analgesia (BPI-SF).
Amato et al. [39]	119 patients with moderate-to-severe cancer-related pain	Observational 60-day study OXN PR ≥ 80 mg/day to manage pain	OXN PR reduced pain intensity ≥30% and the mean number of daily breakthrough pain episodes from baseline BFI; reduced the proportion of patients receiving laxatives; and decreased number of patients reporting nausea, vomiting, or OIC.
Yu et al. [44]	232 moderate-to-severe cancer pain	BPI-SF average pain was comparable after 4 weeks between OXN PR and OXY PR	BFI reduction after 1 week in OXN PR group. Laxative use was similar between the two groups. Average daily dose and percentage of patients taking rescue analgesia were. Fewer adverse events were reported in the OXN PR group.

## Data Availability

No new data were created or analyzed in this study. Data sharing is not applicable to this article.
